# Digital remote monitoring of people with multiple sclerosis

**DOI:** 10.3389/fimmu.2025.1514813

**Published:** 2025-02-28

**Authors:** Michelangelo Dini, Giancarlo Comi, Letizia Leocani

**Affiliations:** ^1^ Faculty of Psychology, Vita-Salute San Raffaele University, Milan, Italy; ^2^ Faculty of Medicine, Experimental Neurophysiology Unit, Institute of Experimental Neurology (INSPE), IRCCS-Scientific Institute San Raffaele, Milan, Italy; ^3^ Department of Neurorehabilitation Sciences, Casa di Cura Igea, Milan, Italy; ^4^ Faculty of Medicine, Vita-Salute San Raffaele University, Milan, Italy

**Keywords:** multiple sclerosis, big data, artificial intelligence, monitoring, review

## Abstract

**Introduction:**

Multiple sclerosis (MS) is a chronic neurodegenerative disease that affects over 2.8 million people globally, leading to significant motor and non-motor symptoms. Effective disease monitoring is critical for improving patient outcomes but is often hindered by the limitations of infrequent clinical assessments. Digital remote monitoring tools leveraging big data and AI offer new opportunities to track symptoms in real time and detect disease progression.

**Methods:**

This narrative review explores recent advancements in digital remote monitoring of motor and non-motor symptoms in MS. We conducted a PubMed search to collect original studies aimed at evaluating the use of AI and/or big data for digital remote monitoring of pwMS. We focus on tools and techniques applied to data from wearable sensors, smartphones, and other connected devices, as well as AI-based methods for the analysis of big data.

**Results:**

Wearable sensors and machine learning algorithms show significant promise in monitoring motor symptoms, such as fall risk and gait disturbances. Many studies have demonstrated their reliability not only in clinical settings and for independent execution of motor assessments by patients, but also for passive monitoring during everyday life. Cognitive monitoring, although less developed, has seen progress with AI-driven tools that automate the scoring of neuropsychological tests and analyse passive keystroke dynamics. However, passive cognitive monitoring is still underdeveloped, compared to monitoring of motor symptoms. Some preliminary evidence suggests that application of AI and big data to other understudied aspects of MS (namely sleep and circadian autonomic patterns) may provide novel insights.

**Conclusion:**

Advances in AI and big data offer exciting possibilities for improving disease management and patient outcomes in MS. Digital remote monitoring has the potential to revolutionize MS care by providing continuous, long-term granular data on both motor and non-motor symptoms. While promising results have been demonstrated, larger-scale studies and more robust validation are needed to fully integrate these tools into clinical practice and generalise their results to the wider MS population.

## Introduction

Multiple Sclerosis (MS) is a chronic, inflammatory, and neurodegenerative disease that affects the central nervous system (CNS). It is estimated that MS impacts ~2.8 million people globally, with a higher prevalence among women (1). MS can cause a wide range of symptoms, depending on the location of lesions across the CNS. Primarily, MS affects sensorimotor functioning, causing vision loss, sensory alterations, walking difficulties, muscle weakness, spasticity, and problems with coordination and balance (2). Additionally, cognitive impairment can be observed in 30-70% of pwMS (3).

The unpredictable nature of the disease, typically characterised by a relapsing-remitting course and by progressive accrual of disability, profoundly affects the quality of life (QoL) of people with MS (pwMS). Furthermore, recent evidence has shown that many pwMS can experience an insidious disease progression even in the absence of relapses (4). Thus, MS poses significant physical, emotional, and socio-economic burdens on individuals and their families (5)​. Accurate disease monitoring is crucial to put in place the best possible treatment plans and reduce the negative impact of the disease on patients’ QoL. Due to organisational and economical limitations of healthcare systems, however, conventional clinical follow-up assessments are generally performed every 6-12 months, or at the time of a relapse. Thus, clinicians are often unable to detect subtle disease progression and/or to capture all relapses, since they need to rely on patients’ recall and infrequent assessments.

The rising adoption of digital health technology in the last decade has sparked an interest in the development, study, and validation of new digital tools for the purpose of monitoring disease progression. Indeed, digital remote monitoring may have the potential to enable longitudinal monitoring of the disease course with a granularity that would otherwise be unobtainable with more costly and less accessible clinical follow-ups (6). A recent European survey found that the vast majority (78%) of patients use commercially-available digital tools (smartphone apps, wearables) to increase awareness of their health, and that 62% of healthcare providers believe that the data obtained from these tools impacts their communication with patients, their understanding of patients’ health state, and their decision-making progress (7). Increasing the adoption of validated digital remote monitoring tools into everyday clinical practice would enable clinicians to access a much larger dataset of quantitative measures which could help them to better understand intra-individual disease trajectories and therefore improve the standard of care for pwMS. Digital remote monitoring can cover a wide range of domains (i.e., motor, cognitive and autonomic functions, psychological wellbeing, disease activity, sleep, diet, etc.), and can be carried out using both active and/or passive monitoring techniques. Active monitoring requires patients to consciously provide information, either via patient-reported questionnaires (e.g., asking patients to rate self-perceived fatigue on a scale 1-10), or by performing objective assessments (e.g., by performing a digitalised cognitive test on their smartphone). Passive monitoring leverages data from smart devices and sensors to enable remote monitoring while patients go about their daily life (e.g., daily steps data from accelerometers in a wearable device, or data from a blood glucose monitor placed on the arm). Active and passive methods can be paired to enhance the quality of digital remote monitoring data (e.g., collecting daily steps data from a participant’s smartphone, which is also used to administered weekly standardized walking tests designed to be performed while carrying the smartphone in the pocket, to measure the distance walked and other data obtained from the smartphone accelerometer and gyroscope).

The definition of ‘big data’ keeps evolving, as continuing technological advancement and increasing adoption of devices able to capture more and more data push the boundaries of “big data”. However, core properties like high volume (i.e., large quantities of data), velocity (i.e., data which are acquired in real-time) and variety (i.e., data which can be either structured or unstructured) are shared across most definitions (8). Other properties like exhaustivity (i.e., the ability to capture an entire system), high resolution (i.e., the ability to collect many datapoints at short intervals), relationality (i.e., the ability to merge different datasets), scalability (i.e., the ability to expand rapidly in size) have also been proposed (8). In general, data which cannot be easily viewed, processed and analysed using traditional statistical methods and which requires *ad-hoc* processing pipelines to produce meaningful insights could be labelled as big data. A consensus definition for big data in health research was proposed by the Health Directorate-General for Research and Innovation of the EU Commission, stating: “*Big Data in health encompasses high volume, high diversity biological, clinical, environmental, and lifestyle information collected from single individuals to large cohorts, in relation to their health and wellness status, at one or several time points*” *(* 9).

In the context of digital remote monitoring of patients, big data can include structured and/or unstructured data from smart devices, wearables, self-monitoring devices, or electronic health records (EHRs) (10). Data from wearables or data recorded passively from smart devices can easily satisfy the “high volume” and “high velocity” criteria of big data. Indeed, using a single tri-axial accelerometer to monitor motor activity of a single individual over 10 hours, with a sampling frequency of 1 Hz, would yield over ~130,000 raw data points, which would need to be processed and aggregated using custom algorithms to derive basic interpretable metrics (e.g., steps/minute), and then further processed to derive more advanced metrics (e.g., time spent performing moderate vs. intense activity). Data from smart devices used to administer active tests is characterized by significantly lower volume and velocity but can become big data in the context of long-term monitoring, especially as digital remote monitoring allows to administer repeated assessments with higher frequency, longer follow-up times, and to larger cohorts, addressing the “scalability” property of big data. In the context of a simple digital cognitive test for which participants need to respond to 50 stimuli, a typical dataset would contain information on response times, actual responses, correctness of each response, metadata (e.g., date, time, type of device, location, device orientation, stimulus order), resulting in >200 datapoints for each testing session. These raw data would also need to be processed and aggregated to derive informative metrics (e.g., mean reaction times). Monitoring 20 patients for 12 months through weekly testing with this simple test would result in the collection of ~50,000 datapoints, with longer and more complex assessments increasing the volume of data acquired exponentially. Data from EHRs typically reaches big data status only when large quantities of clinical data are collected for a large number of patients, either longitudinally in a single centre or cross-sectionally through multicentre collaborations. EHRs data also fits the “exhaustivity” property of big data, as they include a wide range of information for each patient (e.g., sociodemographic, clinical, imaging, pharmacological). Another way that EHRs data can fit the criteria for big data is linked to recent developments in Artificial Intelligence (AI) applied to processing and aggregating of unstructured text data, which could enable to start analysing large quantities of unstructured data present in EHRs (e.g., medical notes) in an automated (or semi-automated) quantitative way, thus greatly expanding the dimensionality of EHR datasets.

**Figure 1:**
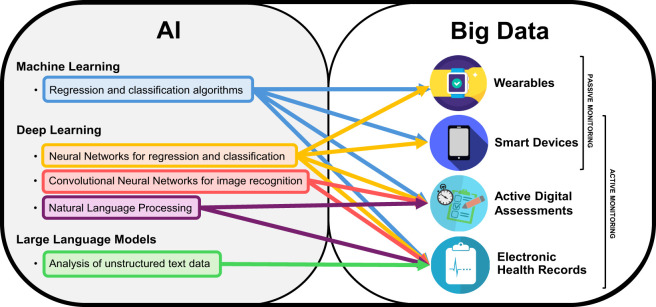
AI and Big Data for digital remote monitoring of MSFigure representing the two sets of Artificial Intelligence (AI) and big data, with specific subfields relevant for the field of digital remote monitoring of people with Multiple Sclerosis. The arrows indicate what type of AI-based analysis is best applicable to different types of big data obtainable from different methods of digital remote monitoring.

AI is a term dating back to the 1950s, when it was coined to represent machines exhibiting features akin to human intelligence (e.g., reasoning, learning, vision) (11). In recent years, this term has transitioned more and more from theory to practice, and many subdivisions of AI have been defined, according to their respective properties and use cases (12). Machine Learning (ML) refers broadly to the use of computational algorithms to learn data patterns to make predictions, and then compare the predictions with the actual outcomes, in order to learn iteratively, thus improving the quality of the predictions based on available data at each iteration. Deep Learning (DL) is an evolution of conventional ML, since it follows the same iterative learning approach to improve predictions. However, it differs from ML in that DL models are built from different consecutive hidden layers of ‘neurons’ (i.e., interconnected processing nodes) which are used to process raw inputs and can be adapted to perform optimally across different specific tasks (i.e., speech recognition, image processing, genomics) (13). One such example are Convolutional Neural Networks (CNN), i.e., DL algorithms built using specific types of connected layers to improve the neural network’s ability to perform image recognition tasks, and have thus found large use in radiology, by allowing automated or semi-automated scoring of CT or MRI scans (14). The ever-increasing worldwide dissemination of computing technology means that more and more data is being collected every day, and the increased computational power available today has made it possible to deploy AI in an increasing number of applications (**Figure 1**).

The aim of this narrative review is to present and discuss recent advancements in the field of digital remote monitoring in MS, with a focus on AI tools and algorithms applied to the analysis of big data from sensors, wearables, smartphones, and other smart devices, as well as data from active digital assessments designed to be performed independently and remotely by patients. Specifically, we aim to discuss how leveraging big data and AI could allow to improve the standard of routine disease monitoring of pwMS across different settings and in different fields, how it could allow researchers to obtain novel insights into specific factors driving disease progression, and what future developments are needed to further advance the state of digital remote monitoring in the future.

## Methods

For this narrative review, we focused our literature search on studies of digital remote monitoring of pwMS using AI and/or big data. This includes studies aimed at validating digital monitoring tools designed to enable active or passive digital remote monitoring of MS symptoms and disease progression. To this aim, we conducted a PubMed search for papers containing the following terms in the title and/or abstract: “multiple sclerosis[Title/Abstract] AND ((‘digital monitor*’[Title/Abstract] OR ‘remote monitor*’[Title/Abstract] OR wearable*[Title/Abstract]) OR (‘artificial intelligence’[Title/Abstract] OR ‘machine learning’[Title/Abstract] OR ‘deep learning’[Title/Abstract]))”. We filtered the search results to only select those published in the last 10 years, i.e., from 1^st^ January 2014 to 1^st^ August 2024.

We then excluded all reviews, meta-analyses, study protocols, opinion papers, editorials. We also excluded all studies where AI or big data where not specifically applied to data from digital remote monitoring or designed to enable it. Therefore, we excluded studies on AI-based processing and analysis of big data from structural (e.g., magnetic resonance) or functional (e.g., positron emission tomography) imaging, robotics-assisted physical rehabilitation, AI-assisted cognitive rehabilitation, AI-based psychological counselling, AI-based analysis of genomics, and those using AI and/or big data to estimate the risk of developing MS or to increase diagnostic accuracy. We also excluded studies of digital remote monitoring in which neither AI nor big data were applicable definitions (i.e., studies aimed at validating the administration of an established clinical test through videoconferencing or other telemedicine approaches, without collection of big data from sensors and/or other electronic devices).

The resulting candidate publications were screened manually by reading the abstracts, to select those who focused on developing and validating data processing and analysis pipelines (including AI) applied or applicable to digital remote data from sensors and/or active remote assessments, as well as those focusing on AI algorithms applied or applicable to the analysis of big data from other sources (e.g., EHRs) to improve the monitoring of disease progression in pwMS.

## Results

Our literature search revealed that the majority of studies on digital remote monitoring of pwMS using AI and big data has focused on the use of wearable sensors to assess and monitor motor symptoms. This is not surprising, as motor deficits are one of the most prevalent and invalidating symptoms of MS (2). Therefore, our review begins by providing a report on studies focused on the motor domain, to evaluate the feasibility and validity of digital remote monitoring of motor functions in real-world clinical applications and highlight issues which still require further development. More recently, other studies have also focused on the need to monitor cognitive symptoms, since they are frequently reported as of the main factors which negatively impact the autonomy and QoL of pwMS (15). We present these studies and discuss the potential added benefits of digital remote monitoring of cognition using AI, compared to the current standard of care, as well as the potential to deploy “big data” to enable passive cognitive monitoring. The use of big data and AI for the digital remote monitoring of other symptoms or domains (e.g., sleep, autonomic functions) or to leverage unstructured big data from EHRs to monitor disease progression are still underrepresented in the MS literature. However, the few studies available to date suggest that their further exploration may yield novel insights which would otherwise be unobtainable by using conventional data acquisition, processing and analysis methods. Therefore, we conclude by presenting the studies available to date, to highlight the potential benefits of these different applications of big data and AI to enhance the remote monitoring of pwMS.

### Motor domain

Many studies in the last 5-10 years have applied big data analysis and AI to the study of motor symptoms, aiming either to enable continuous passive monitoring, validate remote active motor tests to be used for frequent remote active monitoring, or leverage sensor data to detect digital biomarkers associated with higher odds of disease worsening or relapsing. The three main areas of interest appear to be falls (including both automatic fall detection using sensor data and identification of risk factors), gait (including both passive monitoring and active instrumented tests which can be performed remotely and independently by pwMS), and activity monitoring during everyday life as a digital biomarker of disability progression. The characteristics of all reviewed studies are summarised in **Table 1**.

**Table 1:** Summary of studies on motor domain.

Study	Year	Sample	Study type	Sensor array	Algorithms used	Aim	Monitored activities
Özdemir et al. (23)	2014	14 HCs	Cross-sectional	6 accelerometers - 1 on head - 1 on chest - 1 on waist - 1 on right wrist - 1 on right thigh - 1 on right ankle	5 ML algorithms to distinguish between normal activity and falls (kNN, LSM, SVM, BDM, DTW) + 1 DL (ANN)	Automated falls detection	Simulated falls in a controlled setting
Casilari et al. (22)	2015	4 HCs	Cross-sectional	1 smartphone with embedded inertial sensors (accelerometer + gyroscope) 1 smartwatch with embedded inertial sensors (accelerometer + gyroscope)	Mix of custom and published threshold-based algorithms for automated fall detection	Automated falls detection	Simulated falls in a controlled setting
Chitnis et al. (27)	2019	23 pwMS	Longitudinal (three visits over 24 weeks, 8 weeks of remote monitoring)	3 multi-sensing devices (acceleration, motion, heart rate, skin impedance, body temperature, light exposure, air pressure) - 1 on chest (day only) - 1 on right wrist (day and night) - 1 on right ankle (day and night)	DL for automated detection of activity type and quantification of time spent during each activity phase	Validation of remote gait analysis	Instrumented structured assessments in a controlled setting (baseline, week 16, week 24) Passive real-life remote monitoring (8 weeks)
Bourke et al. (30)	2020	76 pwMS 25 HCs	Longitudinal (24 weeks of remote instrumented testing)	1 smartphone with embedded inertial sensors (accelerometer + gyroscope)	Custom algorithms for automated extraction of gait parameters	Validation of remote gait analysis	Instrumented structured assessments performed remotely and autonomously (1/week for 24 weeks)
Atrsaei et al. (28)	2021	35 pwMS	Cross-sectional	3 inertial sensors (accelerometer + gyroscope) - 1 on lower back - 2 on feet (right and left, used only as validation reference)	Custom threshold-based algorithm based on gait speed from multi-sensor data	Validation of remote gait analysis	Instrumented structured assessments in a controlled setting Instrumented structured assessments performed remotely and autonomously (50% of participants)
					ML (naïve Bayes classifier)	Automated walking bouts detection	Passive real-life remote monitoring for at least 6 hours (50% of participants)
Delahaye et al. (33)	2021	18 pwMS	Cross-sectional	1 wearable GPS receiver, placed on the right shoulder	Custom processing and aggregation pipeline for GPS and altitude data; threshold-based algorithm for walking bout detection	Validation of remote gait analysis	Instrumented structured tests in a controlled setting
Meyer et al. (19)	2021	37 pwMS	Retrospective	2 accelerometers - 1 below clavicle - 1 on right thigh 5 inertial sensors (accelerometer + gyroscope) - 1 on lower sternum - 1 on lower back - 1 on belt line - 2 on shanks (right and left)	Fully automated algorithm for data processing; DL for automatic activity detection (Bidirectional Long ShortTerm Memory)	Fall risk estimation	One-minute walking trial at home
Mosquera-Lopez et al. (20)	2021	25 pwMS	Longitudinal (8 weeks of continuous monitoring)	Wireless time-of-flight home beacons, paired with a wearable smart tag (worn either on the trunk or in the pocket) with embedded accelerometer	Fully automated algorithm for data processing; DL for automatic falls detection (neural network auto-encoder + hyper-ensemble of RFs)	Automated falls detection	Passive real-life remote monitoring
Block et al. (35)	2022	94 pwMS	Longitudinal (12 months of continuous monitoring)	Commercial smart band (Fitbit Flex), including inertial sensors and sleep tracking capabilities	ML (3-compartment GMM)	Automated detection of activity states and behaviour patterns	Passive real-life remote monitoring
Creagh et al. (37)	2022	52 pwMS 24 HCs	Longitudinal (24 weeks of remote instrumented testing)	1 smartphone with embedded inertial sensors (accelerometer + gyroscope) 1 smartwatch with embedded inertial sensors (accelerometer + gyroscope)	CNN applied to raw accelerometer data	Disease severity estimation	Instrumented structured assessments performed remotely and autonomously (1/week for 24 weeks)
Salomon et al. (36)	2022	132 pwMS 90 HCs	Longitudinal (1 week of continuous monitoring)	1 wearable accelerometer, placed on the lower back	Custom algorithms for automated detection of activity fragmentation, circadian and fractal patterns	Automated detection of activity states and behaviour patterns	Passive real-life remote monitoring
Sun et al. (31)	2022	337 pwMS	Longitudinal (10 months of continuous monitoring)	1 commercial smart band (Fitbit Charge 2 or Fitbit Charge 3), including inertial sensors, heart rate and sleep tracking capabilities	Fully automated algorithm for data processing; ML regression (RF, GBT, EN) to predict 6MWT performance	Validation of remote gait analysis	Passive real-life remote monitoring
Tulipani et al. (17)	2022	37pwMS	Cross-sectional	2 accelerometers - 1 on chest - 1 on thigh	Fully automated algorithm for data processing; DL for automatic activity detection (Long Short-Term Memory)	Fall risk estimation	Sit-to-stand transitions in everyday life Standardised sit-to-stand task in the lab
Granja Domínguez et al. (32)	2023	205 pwMS	Cross-sectional	2 insoles with inertial sensors (accelerometer + gyroscope) and pressure sensors	Custom algorithm for automated calculation of gait parameters	Validation of remote gait analysis	Instrumented structured tests in a controlled setting
Kushner et al. (21)	2023	25 pwMS	Longitudinal (8 weeks of continuous monitoring)	Wireless time-of-flight home beacons, paired with a wearable smart tag (worn either on the trunk or in the pocket) with embedded accelerometer	DL for automatic fall detection (Long Short-Term Memory); ML for room detection (kNN)	Automated falls detection	Passive real-life remote monitoring
Salis et al. (26)	2023	128 participants, both HCs and people with mobility issues (20 pwMS)	Cross-sectional	3 inertial sensors (accelerometer + gyroscope) - 2 on feet (right and left) - 1 on lower back 2 time-of-flight infrared sensors placed on ankles (right and left) 2 pressure insoles	Custom threshold-based algorithms for automated walking bout detection	Automated walking bouts detection	Passive real-life remote monitoring (2.5 hours)
					Custom algorithm for automated gait analysis based on integration of multi-sensor data	Validation of remote gait analysis	Structured tests in a controlled setting Simulated activities of daily living in a controlled setting
Stavropoulos et al. (34)	2023	2 pwMS	Proof-of-concept	Commercial smart band (Fitbit Charge 3), including inertial sensors, heart rate and sleep tracking capabilities	Knowledge graphs	Automated detection of activity states and behaviour patterns	Passive real-life remote monitoring
Vandyk et al. (18)	2023	23 pwMS	Longitudinal (6 weeks of continuous monitoring)	3 accelerometers and surface biopotential readers - 1 on left upper chest - 2 on thighs (right and left)	Fully automated algorithm for data processing; DL for automatic activity detection (Long Short-Term Memory)	Fall risk estimation	Passive real-life remote monitoring Sit-to-stand transitions in everyday life (detected automatically)
Kirk et al. (29)	2024	97 participants, both HCs and people with mobility issues (13 pwMS)	Cross-sectional	1 experimental inertial sensor (accelerometer + gyroscope) placed on the lower back 3 reference inertial sensors (accelerometer + gyroscope) - 2 on feet (right and left) - 1 on lower back 2 time-of-flight infrared sensors placed on ankles (right and left) 2 pressure insoles	Mix of custom and ML-based algorithms for automatic gait detection and calculation of gait speed	Validation of remote gait analysis	Instrumented structured tests in a controlled setting Simulated activities of daily living in a controlled setting Passive real-life remote monitoring (2.5 hours)

Articles are listed based on year of publication (in ascending order). 6MWT, 6-Minutes Walking Test; ANN, Artificial Neural Network; BDM, Bayesian Decision making; CNN, Convolutional Neural Network; DL, Deep Learning; DTW, Dynamic Time Warping; EN, Elastic Net; GBT, Gradient Boosted Trees; HCs, Healthy Controls; kNN, k-Nearest Neighbours; LSM, Least Squares Method; ML, Machine Learning; pwMS, people with Multiple Sclerosis; RF, Random Forest; SVM, Support Vector Machine.

#### Risk of falls

Falls are a major health concern for pwMS, as over 50% of them are estimated to experience at least one fall in a 6-month period, of which half result in injury (16). Continuous remote monitoring of pwMS in real-life environments and automatic falls detection has the potential to increase the detection rate of falls in everyday life, allowing a more precise monitoring of clinical progression. Moreover, it could help identify specific risk factors and consequently develop prevention strategies.

Tulipani et al. (17) investigated the ability to predict fall risk in 37 pwMS wearing a chest and a thigh sensor during sit-stand transitions of daily life and during a standardised sit-stand task in the clinic. Using reported falls in the previous 6 months to dychotomize participants in “fallers” or “non fallers”, they evaluated the ability of sensor data to correctly classify patients in either class. Sit-to-stand transitions in daily life were detected using a DL (long Short-Term Memory) algorithm tuned to detect activity states, which allowed them to select only sensor data from periods of transition from the “sitting” to the “standing” state. Using Receiver Operating Characteristics (ROC) analysis, the best predictor of high fall risk in their study was a chest acceleration metric recorded during execution of the sit-stand task in the clinic (Area Under the Curve [AUC]= 0.89). The best performing sensor metric during the real-life task execution, i.e., average sit-stand time, had slightly lower predictive power (AUC= 0.81). Their results suggest that conventional sensor metrics (e.g., acceleration, total time of execution) may provide useful insights into the fall risk of pwMS, although with reduced accuracy, compared to instrumented functional assessments performed in the clinic. The same research group recently published a longitudinal study (18), with the aim of extending the analysis of sit-stand performance to longitudinal remote monitoring. They recruited 23 pwMS and monitored them for six weeks, using three wearable sensors worn for all hours of the day (one on the left upper chest, two on the thighs) to record acceleration and surface biopotentials. Furthermore, they applied DL analysis to detect periods of sit-standing transitions. The algorithm identified different fatigue and instability phenotypes which were predictive of fall risk. They also observed that stability tended to decline over the course of the day, providing interesting quantitative insights into daily fluctuations of motor performance. Taken together, these results suggest that DL algorithms may enable to reliably identify activity states remotely and during everyday life, thus allowing to contextualise motor features obtained by the analysis of big data collected continuously from sensors. This is particularly interesting, since novel insights could be obtained by investigating some motor features (e.g., stability) during specific activity states of interest (e.g., sit-to-stand transitions), rather than across the entire range of daily activity states, which would be unfeasible if activity states had to be observed by an examiner or reported by the patient.

DL algorithms were also implemented retrospectively, to detect patients who had a positive recent history of falls (in the previous six months), by leveraging accelerometer data from sensors placed on the sternum, lower back, thigh, and shanks during a one-minute walking task in the clinic (19). This study found that a bidirectional long short-term memory neural network could be used to automatically identify and analyse sensor data from 1-minute walking tests performed remotely and autonomously by pwMS, and identified pwMS who had previously fallen with high accuracy (ROC AUC= 0.88). Notably, this DL algorithm trained on raw sensor data significantly outperformed the classification accuracy of neurologist-administered measures and patient-reported outcome measures, as well as conventional statistical analyses and other traditional ML models (logistic regression, k-nearest neighbours, support vector machine, decision tree) based on conventional aggregate spatiotemporal gait parameters (e.g., average speed). This suggests that AI can leverage big data to capture nonlinear relationships and motor phenotypes associated with an increased risk of falls which are not detected through conventional clinical exams or basic aggregate statistics.

Another key application of big data is the automatic detection of real-world falls in freely moving patients through sensors from wearables and/or smartphones. Mosquera-Lopez et al. (20) developed an algorithm which detects possible falls by combining acceleration and movement features recorded by wearable sensors connected to wireless beacons placed throughout the home. As fall detection was performed in a fully unsupervised way, accuracy of the detection pipeline was tested using 10-fold cross-validation (CV). This system proved highly accurate in detecting falls (sensitivity= 92%, specificity= 98%), producing 0.65 false alarms per day, which translates to roughly 2-3 false alarms per week. However, due to the small sample size and relatively short monitoring time, their dataset was highly imbalanced, with only 270 seconds of fall data compared to over 2,000,000 seconds of total data. In a more recent study (21), the same researchers conducted a secondary analysis of the same dataset, to investigate the relationship between mobility measures (including both movement metrics and location data) and risk of falls in pwMS. They found that half of falls occurred while walking, and that participants were sedentary for most of the time spent at home (>95%). Interestingly, they were able to observe that almost one third (28%) of falls occurred within one second of gait initiation, thus providing quantitative data to highlight the critical role of gait initiation in determining fall risk during everyday life. These results are promising, although the feasibility of this tracking method is obviously lower than that of monitoring devices which do not require altering/adapting the home environment of patients, which could hinder its applicability for real-life long-term monitoring of pwMS. Moreover, such systems cannot be used to assess motor performance in everyday life settings other than patients’ homes (e.g., the workplace), limiting the generalizability of their findings. Further studies with much larger samples and longer monitoring durations are required to assess the true feasibility of this monitoring approach, as well as its validity and reliability for real-life clinical applications.

Increasing the range of possible applications of digital remote monitoring is key, to enable monitoring of motor functioning in a more ecological way, which would also allow extend this possibility to a wider range of pwMS. Therefore, more and more studies have tried to leverage commercially available smart devices for remote data collection, as their widespread availability could greatly extend the reach of digital remote monitoring, compared to more experimental and multi-device approaches. A pilot study (22) investigated the ability of a commercially available smartphone and smartwatch to automatically detect falls in an experimental environment, in which healthy controls (HCs) performed a set of simulated falls. Using an experimental setting in which participants performed simulated falls, they were able to directly observe the number of false positives and false negatives produced by the fall detection algorithm, from which they calculated sensitivity and specificity. They found that the joint use of smartphone and smartwatch improved the specificity of all analysed algorithms by a range of 5-15%, compared to smartphone- or smartwatch-only detection, although the issue of false positives alarms remained, as denoted by several false alarms raised during 24h of continuous monitoring. Moreover, the extremely small sample size (N = 4) significantly limits the generalisability of their results. Another study (23) investigated automatic fall detection through a system of tri-axial sensors fitted to six different body parts (head, chest, waist, right wrist, right thigh, right ankle) of HCs performing a standardised set of voluntary falls in an experimental setting. Through ML analyses they were able to reach values >99% for accuracy, sensitivity, and specificity. However, it must be stressed that this result was again observed in a small sample of HCs, performing standardised falls in a controlled setting. Perhaps even more importantly, such a complex sensor array would likely be unfeasible for everyday real-life monitoring of pwMS. It should be noted that studies wishing to evaluate automatic falls detection accuracy through direct observation (i.e., through simulated falls experimental paradigms) are inherently limited, since having pwMS or people with other chronic health conditions performing simulated falls would pose evident ethical and safety issues. Crucially, this questions the ecological validity of fall detection algorithms validated on young healthy participants. Further research is needed to determine the feasibility and validity of automated fall detection through smartphone and/or wearable data in pwMS and in real-life scenarios.

#### Gait analysis

Gait disturbances are common in MS, they can present in the early disease stages, and significantly affect QoL by reducing autonomy and impacting negatively on socio-economic status (24). Instrumented assessments of gait are well documented (25) but, until recently, have largely relied on sophisticated lab-based assessments which are costly, cumbersome, and can fail to capture the true walking performance of pwMS in real-life environments. Consequently, most research to date has focused on validating wearable data recorded during laboratory experiments in which participants perform a mix of structured tests and simulated real-life activities. Only recently, researchers have begun leveraging big data gathered from wearables during everyday life to estimate gait parameters of pwMS, or to validate such monitoring devices with a mixed study procedure including both lab-based and remote-based data collection.

Salis et al. (26) validated a multi-sensor system designed to allow real-world monitoring (three inertial sensors, two plantar pressure insoles, and two distance sensors) in 128 participants with different pathologies (including 20 pwMS) who performed a mix of structured tests (e.g., Timed-Up and Go) and simulated activities (e.g., setting the table for dinner). They compared data from the wearable sensors with data from a stereophotogrammetry system, which served as reference. They used intraclass correlation coefficients (ICC) to assess reliability, which can be considered excellent when ICC > 0.90, good when 0.75 < ICC < 0.90, moderate when 0-5 < ICC < 0.75, and poor when ICC < 0.50. The reliability of the wearable system was excellent for structured tests, with ICC values >0.95, while it decreased slightly for simulated activities of everyday life (ICCs between 0.69-0.98). They also evaluated the feasibility of this wearable system for real-life use by recording 2.5 hours of unsupervised activity and reported that the system was well accepted, without major technical or usability issues. However, it must be noted that the real-world part of this study included only 20 healthy young adults and lasted a short time. Further real-life feasibility and acceptability studies with much longer monitoring periods are therefore definitely needed to derive any meaningful conclusions on real world long-term feasibility.

Chitnis et al. (27) collected data remotely from 23 pwMS wearing three sensors (placed on wrist, ankle, and sternum) for eight weeks during real-world daily activities. They designed a workflow for the classification of unstructured raw sensor data, using a DL classifier to distinguish activity periods (i.e., idle, walking, running). Then, they selected only the activity segments classified as “walking” to derive mobility features. Several features extracted from real-world walking bouts (i.e., stance time, swing time, mobility activity time, turning velocity) correlated with gold-standard clinical scales like the Expanded Disability Status Scale (EDSS) and the Multiple Sclerosis Functional Composite (MSFC) and standardised walking tests performed in the clinic (Timed 25-Foot Walk [T25FW]).

While multiple wearable sensors undoubtedly afford a higher degree of precision and provide more data to extract spatiotemporal gait parameters, compared to a single wearable sensor, one must also consider the feasibility of such approaches for longitudinal remote monitoring. Indeed, using multiple sensors imposes higher costs and is more burdensome for patients and researchers alike. This issue grows exponentially with longer follow-up times, limiting the ability to study long-term trends and patterns of motor function in pwMS. More specifically, compared to wearing sensors on multiple body parts, using a single sensor facilitates monitoring in a wider variety of daily life situations (e.g., in public), enhancing the ecological validity of data thus collected. Therefore, some researchers have begun to evaluate the validity of data obtained from a single wearable sensor, which could prove more economical and easier to use, therefore allowing larger studies with longer follow-ups.

Atrsaei et al. (28) developed and validated a ML-based gait estimation approach to predict gait speed and detect waling bouts using a single sensor on the lower back. They recruited 35 pwMS, who performed walking tests in the clinic and at home. and found that reference values obtained from sensors on both feet correlated strongly with gait speed estimated from the sensor on the lower back during a walking test in the clinic (*r* = 0.96) and at home (*r* = 0.95); gait speed during daily activities at home were also strongly correlated with reference values recorded in the clinic (*r* = 0.89). These results show that not only using a single sensor on the back approximates reference values extremely well for walking tests performed in the clinic, but is also able to provide accurate estimation based on real everyday activities. They also tested a ML-based algorithm (naïve Bayes classifier) for automated walking bouts detection and used leave-one-out CV to evaluate its accuracy, using only digital remote data collected during unsupervised daily life activities. The ML-based walking bout detection had high accuracy (96.4%) in detecting walking bouts remotely, during everyday life. Although the authors reported analysing >300 hours of daily activity measurements, the small sample size significantly limits the generalizability of these promising results obtained using a single sensor.

A similar approach (single sensor worn on the lower back; in the clinic and during 2.5 hours of real-world activities) was adopted by a European multicentric study (MOBILISE-D) on N=97 participants with different medical conditions (including 13 pwMS) (29). Reliability was considered good-to-excellent in the clinic (ICC range= 0.79-0.91) and moderate-to-good (ICC range= 0.57-0.88) in real-world activities, compared to a multisensory reference system which included pressure insoles. Although the reliability of the system was lower in the real-world scenario, it was still deemed to remain within a usable range. Predictably, this study found that walking bout duration affected the accuracy of gait speed estimation, with shorter bouts yielding less accurate estimates. It is therefore possible that further studies with more data at the intra-individual level may yield higher accuracy.

Aiming to further explore the use of devices which could be accessible to larger proportions of the population, Bourke et al. (30) analysed gait parameters recorded by a waist-worn smartphone with built-in accelerometer during a two-minute walking test performed remotely and independently by 76 pwMS and 25 HCs over 24 weeks. The test-retest reliability across consecutive pairs of testing sessions was either excellent or good-to-excellent for 58/92 gait parameters in pwMS, and 29/92 in HCs, indicating higher variability in healthy persons across consecutive test sessions. These results suggest that remote sensor data recorded during active walking tests, using only a waist-worn smartphone, has comparable reliability to sensor data from clinical assessments. This encourages further research, as it could enable a much wider diffusion of instrumented remote walking assessments tanks to the ever-increasing availability of smartphones and wearables, thus expanding the reach of gait monitoring to those with reduced access to clinical services. However, this study involved mainly people with relapsing-remitting MS (RRMS), and only data from 62 participants (51 pwMS, 11 HCs) was used for the analyses (the authors did not explicitly state the reason for excluding almost 40% of the initial sample size). Therefore, further studies with larger sample sizes and more rigorous reporting are needed to establish the feasibility and validity of using smartphone-based sensor data as an endpoint in clinical trials and for real-life clinical monitoring.

All the studies examined so far have been conducted on small samples, and their results cannot therefore be generalised to the wider population of pwMS. The large volume of data obtained through wearable sensors and the high costs associated with specialised sensors has greatly limited the ability of researchers to conduct studies on large samples and with adequately long follow-ups, as evidenced by the studies discussed so far. Multicentric studies on larger samples of pwMS, however, are needed to derive more reliable insights on the validity, reliability, and feasibility of digital remote monitoring tools. As part of the RADAR-CNS initiative, Sun et al. (31), monitored an European cohort (from Italy, Spain, and Denmark) of 337 pwMS over an average duration of 10 months using a commercial wearable (Fitbit). They analysed real-world steps data and applied correlation-based feature selection to select the most relevant features and tested the ability of different ML regression algorithms (random forest, gradient boosted trees, and elastic net) to estimate 6 Minutes Walking Test (6MWT) performance in the clinic by using digital remote monitoring data collected during everyday life. Results show that minute-level features were more predictive than day-level features. Interestingly, they also noted that upper bound statistics (e.g., 90^th^ percentile of minute-level step count) were more strongly related to clinical test scores, indicating that the average performance in clinical gait tests may reflect the upper portions of the distribution of real-life gait abilities. This insight is particularly valuable, as it could mean that the impression of motor functioning that a clinician gets from a patient performing a walking test in the clinic may be an overestimation of their actual day-to-day average motor performance. The accuracy of 6MWT score estimation was quite low, reinforcing the idea that walking performance of pwMS could differ significantly between real life and clinical testing. These findings demonstrate that, in addition to allowing digital remote monitoring, leveraging data from wearables collected during everyday life can provide insights that would not be obtainable through conventional study paradigms, thus improving our understanding of the true validity of gold-standard and widely used clinical tests.

Another study with a large sample size (32) (N = 205 pwMS) focused on validating gait parameters (velocity, ambulation time, cadence, stride length) estimated trough sensor data from connected insoles with pressure and motion sensors, compared to a classic lab-based reference system based on pressure plates. They showed strong concordance between the two systems for gait velocity (ICCs > 0.83), ambulation time (ICC = 0.93), and cadence (ICCs > 0.90), whereas stride length showed poor concordance (ICC = 0.30). Sensorised foot insoles allow continuous data collection in everyday life without requiring visible devices, which could cause stigma or discomfort to some patients. Therefore, this large study provides valuable data on the validity of this gait monitoring device, which may prove particularly useful for patients which are unwilling and/or unable to wear visible devices such as smartwatches or body-mounted sensors. However, one key limitation is the compatibility of insoles with different shoe types, and the need to switch the insoles when changing shoes and when recharging, which could prove burdensome for patients in the long term, and could lead to missing data for extended periods of time or in some specific settings (e.g., while wearing slippers at home).

Whereas most of the literature to date has focused on obtaining gait parameters from accelerometers, Delahaye et al. (33) investigated gait parameters derived from a wearable sensor with integrated Global Positioning System (GPS). Validating GPS-derived walking speed and distance metrics may potentially enable to implement remote monitoring via commercially available and non-wearable devices (e.g., smartphones), thus removing the need for specially designed wearable sensors which may be perceived as cumbersome or that patients may be embarrassed to wear in public. The authors recruited a small convenience sample (N = 18) of pwMS who performed the 6MWT and an outdoor walking session at usual pace (up to 60 minutes). By integrating GPS and altitude data, they were able to measure gait parameters and associate them with variations in the terrain conformation, which could not only allow to better understand variability in motor activity observed through digital remote monitoring, but may also be used to standardize future studies on outdoor walking performance across different centres and countries, They found that walking speed during an outdoor walking session was significantly correlated with 6MWT performance measured in the clinic, whereas maximum walked distance was not. They also noted that 40% of participants did not reach their maximum walking distance during the first walking bout, but on subsequent ones. This suggests that the first stint of a walking task (as is the case for clinical walking tests) may not necessarily yield the best performance. Once again, one can appreciate how real-world motor data collected remotely and digitally was able to provide novel insights which enhance our understanding of the validity of testing procedures performed routinely in clinical or research settings. However, only 12 participants had valid GPS data, which means that GPS data could not be analysed for one third of participants. Therefore, more studies are needed to validate GPS-derived measures, and several technical limitations must be addressed, such as the accuracy of GPS-calculated walked distance for shorter walking bouts, or its accuracy in different environments and settings.

#### Activity monitoring

Data from wearable sensors may be used to characterise patients not only in terms of their raw quantitative performance metrics (e.g. daily step count), but to infer activity states and behavioural patterns which may be associated with clinical features and/or impact disease progression. This may be done either using knowledge-based frameworks or with a data-driven approach, providing both researchers and clinicians with more readily interpretable outcome measures. Moreover, characterising activity states may enhance the informative value of raw quantitative measures (e.g., by differentiating between steps counted during a light walk or during an intense run).

An example of the knowledge-based approach has been proposed by Stavropoulos et al. (34), who showcased a framework using *a priori* semantic rules to model “problem labels” which could be quickly and easily understood by clinicians and provide added value to raw quantitative data. As an example, “Steps < 500 & Heart Rate < 100 for duration > 800” was a rule used to determine an instance of “Lack of Movement”. They then reported the example of a patient for which “Lack of Movement” instances appeared sporadically in the first months of remote monitoring and intensified in time, ultimately occurring almost every day in the last months. This provides a simple and effective way for clinicians to monitor potential risk factors and/or indices of disease worsening without necessarily having to analyse raw data, which may be cumbersome or outright unfeasible depending on the resources of different healthcare centres. However, frameworks based on *a priori* rules strongly rely on the goodness of their assumptions, and the validity of their output must be carefully assessed with *ad-hoc* studies implementing baseline and follow-up clinical assessments to provide quantitative measures of disease progression.

Block et al. (35) adopted a data-driven approach to characterize walking activity, based on minute-to-minute steps data from 94 pwMS who wore a Fitbit continuously for 1 year. They applied an unsupervised ML clustering algorithm (3-compartment Gaussian Mixture Model) to detect the proportion of three levels of activity (low, moderate, high) based on individual participants’ steps data, and then evaluated associations with clinical parameters (walking tests, EDSS scores) and patient-reported outcomes. The detected activity levels correlated more strongly with clinical and patient-reported outcomes, compared to raw step count, and the combination of raw steps data and activity levels outperformed both individual metrics. This suggests that the qualitative aspect of steps data plays a pivotal role in predicting key clinical outcomes such as EDSS score. While we can expect patients with lower disability to be more active overall, leveraging AI algorithms to continuously and automatically evaluate the proportion of time spent in low- or high-intensity walking may enable to differentiate two patients which would appear identical if one were to look only at basic aggregate statistics like step count. Indeed, 1000 steps could be performed while doing house chores over 1 hour, or during a short but intense 5-minute walk, two different activities which cannot be accurately distinguished by examining step count alone.

Salomon et al. (36) collected data from 132 pwMS and 90 HCs wearing an accelerometer placed on the lower back for seven days, aiming to uncover daily-living rest-activity fragmentation patterns, circadian rhythms, and fractal regulation parameters. Results showed that pwMS had a more fragmented activity behaviour (likely indicating a greater need for pauses when carrying out prolonged physical activity) and lower amplitude in circadian changes of daily activity (i.e., the difference in activity levels between the five most and least active hours of the day) than HCs. Moreover, both circadian and fragmentation measures were associated with disability severity, as measured by EDSS score. Although a simple general metric like total physical activity remained the strongest discriminator between pwMS and HCs, this study found that incorporating more sophisticated metrics like fragmentation patterns and circadian rhythms detection improved the ability to differentiate between patients and HCs, and between patients with low vs. high disability. This was a cross-sectional study, and therefore could not provide any info on the predictive value of these features on disability progression or relapse risk. However, it is possible that circadian rhythms and fragmentation patterns could also provide novel insights on disease progression (e.g., a patient maintaining the same overall level of activity, but with increased fragmentation due to requiring more frequent rest). Further studies are needed to establish the utility of more advanced activity measured for real life monitoring of pwMS, with specific emphasis on their ability to predict relapse and/or disease progression.

Creagh et al. (37) also adopted a data-driven approach, analysing raw sensor data (smartphone + smartwatch) of 97 participants (24 HCs, 52 pwMS with mild disease severity, 21 pwMS with moderate disease severity) who performed a daily two-minutes walking test remotely for 24 weeks. Raw sensor data were analysed with a deep CNN pre-trained on an open-source human activity recognition dataset, to calculate a continuous quantitative measure of disease severity at each timepoint. Average disease severity across all timepoints correlated strongly with EDSS score. More interestingly, longitudinal disease severity trends were found to be associated with self-reported relapses. These preliminary results suggest that a continuous quantitative measure of disease severity may be more sensitive to change than the EDSS, and that it could also allow to detect trend changes in quasi-real time, which could potentially enable researchers and clinicians to detect relapses and shifts to progressive MS more efficiently. However, significant limitations such as adherence to frequent active testing and reliability of remote tests must be addressed, before such measures can be effectively implemented in everyday clinical practice. Indeed, the authors report that adherence was highly variable across participants, as participants with mild MS showed higher adherence than those with moderate MS and HCs. Moreover, adherence decreased linearly for all subgroups at later timepoints and, in some cases, in concomitance with the onset of reported relapses, as patients stopped performing the walking tests once they began experiencing a significant worsening of motor function happening. These preliminary findings suggest the need to evaluate adherence to digital remote monitoring via active testing not only as a function of time, but also by uncovering potential associations with sociodemographic data (e.g., economic status, age), clinical features (e.g., cognitive impairment, depressive symptoms), or disease progression (e.g., patients becoming wheelchair-bound).

### Cognitive domain

The use of AI and big data for monitoring cognitive function in pwMS has seen significantly less development, compared to the monitoring of motor function. This is likely because evaluating cognitive processes relies much more explicitly on active testing, and it is therefore more laborious to obtain large amounts of data. Indeed, a wearable sensor can detect thousands of datapoints for many motor features passively, just by being worn during everyday activities. The same approach cannot be easily applied to cognitive processes like memory or information processing speed, which are latent variables which need to be evaluated through specifically designed tasks. This significantly limits the ability of researchers to deploy big data to study cognition in MS. Nevertheless, some recent efforts have been made to integrate AI and big data in this field, and their results point to some interesting avenues for future research. The characteristics of all reviewed studies are summarised in **Table 2**.

**Table 2:** Summary of studies on cognitive domain.

Study	Year	Sample	Study type	Cognitive domain	Algorithms used	Aim	Type of monitoring
Birchmeier et al. (38)	2019	135 pwMS	Cross-sectional	Visuospatial learning	CNN for image classification task	Validation of automated test scoring	Active testing
Birchmeier et al. (40)	2020	294 pwMS	Cross-sectional	Visuospatial learning	CNN for image classification task	Validation of automated test scoring	Active testing
Petilli et al. (41)	2021	35 HCs	Cross-sectional	Visuo-constructional ability and visuospatial memory	Custom algorithm for image preprocessing, segmentation and scoring of spatial, procedural and kinematic features	Enhancing the informative value of conventional tests	Active testing
Khaligh-Razavi et al. (42)	2020	91 pwMS 83 HCs	Cross-sectional	Information processing speed	ML multinomial logistic regression	Validation of digital test for autonomous and remote use	Active testing
Lam et al. (45)	2021	102 pwMS 24 HCs	Cross-sectional	–	Custom algorithm for processing and feature extraction from single-keystroke level datapoints	Validation of keystroke dynamic for monitoring of cognition	Passive monitoring
Lam et al. (46)	2022	102 pwMS	Longitudinal (12 months of continuous monitoring and clinical follow-ups every 3 months)	–	Clustering and PCA of features extracted from keystroke data; LMM to evaluate associations with cognitive outcomes	Validation of keystroke dynamic for monitoring of cognition	Passive monitoring

Articles are listed based on year of publication (in ascending order). CNN, Convolutional neural Network; HCs, Healthy Controls; LMM, Linear Mixed Models; ML, Machine Learning; PCA, Principal Component Analysis; pwMS, people with Multiple Sclerosis.

#### Active monitoring

Most efforts have been focused on developing digital versions of established neuropsychological tests, with the aim of enabling automated administration and scoring, thus enabling remote administration and freeing up time for clinicians. In such cases, AI can provide novel ways to automate test administration and scoring, whereas big data has been mainly viewed in the context of granular digital test metrics which would be unfeasible to record manually, but which could enhance the information obtained from the execution of a test, compared to conventional scores.

Birchmeier et al. (38) aimed to digitize the Brief Visuospatial Memory Test – Revised (BVMT-R), a visuospatial learning test which is considered one of the gold-standard cognitive tests in MS (39). Scoring this test is a time-consuming semi-quantitative procedure which requires trained healthcare professionals to evaluate the shape and position of 18 drawings, assigning a score ranging 0-2 to each drawing, and then calculating the final total test score. The authors tested the ability of a CNN to automatically score patients’ drawings, and compared its accuracy to clinician ratings, using a validation sample of 135 patients (for a total of 624 drawings). The CNN achieved a good accuracy for perfect or completely wrong drawings (i.e., those scored either 0 or 2 by human raters), while the accuracy for partially wrong drawings (i.e., those scored as 1 by human raters) was unsatisfactory (57%). This suggests that CNNs may not yet substitute clinicians and enable fully automated scoring, especially for drawings which present only slight inaccuracies and are therefore trickier to score, as they require higher-level decision making than what AI can provide as of today. However, AI-based predictions may be implemented to provide preliminary recommendations, thus enabling faster scoring by human raters and reducing organisational burdens. In a subsequent study (40) with a larger validation sample size (1525 drawings), the authors observed that automated ratings matched with 72% of ratings from one neuropsychologist, and with 79% of ratings from another neuropsychologist. Interestingly, when comparing the ratings given by the two neuropsychologists, they observed an agreement in 82% of cases, highlighting the inherent unreliability of such semi-quantitative scoring protocols. This highlights the need to carefully consider the outcome metrics of AI validation studies, especially for semi-quantitative ratings, not only for cognitive tests, but also for other applications (e.g., MRI lesions counting). Indeed, aiming for 100% accuracy, especially while using a small number of human raters as reference may not be the ideal method. In such cases, reaching 100% accuracy could either be impossible, or lead to overfitting (i.e., training the AI algorithm to become an essential copy of that particular group of raters, which lead to poor generalizability and reliability). Conversely, an AI-based support-decision system may allow to increase inter-rater reliability, as AI-based criteria should hypothetically be more consistent that human raters, although *ad-hoc* studies are needed to support this hypothesis.

Another study focused on automated scoring of visuospatial tests (41), with the aim of providing more varied and detailed performance metrics, compared to the conventional scoring procedure, which only yields a single score indicating overall accuracy. They developed a tablet-based version of the Rey Complex Figure copy task, a visuo-constructive and visuospatial memory task which relies on semi-quantitative scoring, similarly to what has been described above. They administered it to 35 HCs and extracted performance indices capturing three different aspects of drawing abilities (spatial, procedural, and kinematic), for which a composite score was also calculated. They showed that automated scoring via CNNs could provide a much richer performance profile, by aggregating large quantities of data which could not be feasibly recorded manually by clinicians administering a test in a clinical setting (e.g., pressure strength, velocity, procedural drawing timeline). This may be very useful for research purposes and may ultimately lead to better classifications of cognitive profiles in MS (i.e., by disentangling the effect of motor, procedural, and visuospatial deficits). Therefore, the potential benefit of automated scoring may not be limited only to reducing test administration and scoring times. Indeed, automated AI-based scoring based on constructional and/or procedural drawing features recorded digitally may ultimately yield higher consistency than current scoring methods based on semi-quantitative ratings made by humans. However, such procedures require a high degree of standardisation; in this study, all participants used the same hardware, and drawings had to be manually screened before AI-based scoring.

Khaligh-Razavi et al. (42) developed a custom computerized image classification task to assess processing speed, and validated it in a sample of 91 pwMS and 83 HCs. The novelty of their approach consists in the embedding of AI (in the form of a ML multinomial logistic regressor) in the testing pipeline, so that their test does not yield a quantitative score, but rather a multi-level prediction on the cognitive status of the examinee, along with its associated predicted probability. This approach aims to predict cognitive status by automatically integrating a multi-dimensional feature set comprised of basic test scores (e.g., classification accuracy),more sophisticated metrics (e.g., intra-trial accuracy over time), and demographic data (e.g., age and education) to produce predictions on cognitive status on a test-by-test basis. By comparing the predictions made by the ML algorithm with cognitive impairment labels based on published cutoff values for gold-standard neuropsychological tests administered in the clinic, they demonstrated excellent discriminant validity for cognitive impairment in MS (AUC = 0.95, sensitivity = 82.9%, specificity = 96.1%). This approach to cognitive testing merits further research, as it may present many significant advantages. For clinical practice, it could reduce time allotted to test administration and scoring, as the test procedure is automated and seamlessly provides a prediction on cognitive status, thus enabling clinicians to dedicate more time to interact with patients and caregivers. For research purposes, an integrated AI data analysis pipeline allows to automatically leverage a larger amount of test performance metrics to derive more detailed insights into the cognitive profile of pwMS. Finally, automated ML-based scoring can leverage consecutively acquired data to continuously upgrade its predictions, likely making it ever more accurate as time progresses and more data is acquired, without the need for repeated validation studies which can be costly and time consuming.

#### Passive monitoring

Passive monitoring of cognitive functions represents an exciting frontier, as it could potentially enable granular long-term monitoring through big data analysis, without the need for patients to allocate time and energy to actively performing cognitive tests. This could increase the feasibility of continuous monitoring over the years, something which is very hard to achieve through active monitoring, where attrition naturally increases as time progresses (43, 44). However, there is still little evidence on what methods could enable valid and reliable passive monitoring of cognitive functioning.

Lam et al. (45) developed a keyboard app for smartphones, which allows to passively track timing-related keystroke features (e.g., latency between successive key presses, hold time, flight time) and correction-based features (e.g., correction duration, pre-correction slowing). They recruited 102 pwMS and 24 HCs, who were monitored passively as they used the keyboard app for 14 days. Results showed weak-to-moderate correlations with clinical disability, cognitive functioning, and upper limbs dexterity, as measured by the gold-standard clinical tests. Moreover, they observed that most timing-related features were significantly different between HCs and pwMS. In a follow-up longitudinal study (46), they monitored 102 pwMS for 12 months, using the keyboard app for passive monitoring and via clinical follow-ups every three months with clinical tests for upper limb dexterity and cognition. To evaluate associations between passive monitoring features and clinical features, they aggregated keystroke data into a cognition score cluster and a fine motor score cluster. They found that the cognition score cluster was significantly associated with cognitive functioning at the group level, but not at the individual level, whereas the fine motor score cluster was significantly associated with upper limb dexterity at both the group and individual level.

In conclusion, the evidence available so far indicates that keystroke dynamics may be used to passively monitor longitudinal upper limb dexterity changes at the intra-individual level, whereas the same cannot be yet said for cognitive changes, suggesting that practice effects of repeated testing may have been a confounding factor. Moreover, the concurrent validity of keystroke dynamics is significantly lower than that of digitalized active cognitive tests (47). This is to be expected, as everyday activities such as typing leverage various sensory, motor, and cognitive processes and are not typically performed as rigorously and precisely as cognitive tasks, therefore introducing more noise. Thus, further research is needed, before keystroke dynamics can be considered an effective and reliable passive monitoring tool for cognition in MS. However, the potential to obtain data on cognitive functioning without requiring conscious effort by patients remains an enticing prospect, since it would allow to eliminate the aforementioned issue of loss to follow-up common to active longitudinal testing, and could provide novel, undiscovered insights on the cognitive functioning of pwMS by truly leveraging big data. One key aspect that should be addressed in the future regards the ethics of collecting keystroke data, as it could theoretically allow to uncover patients’ sensitive information (passwords, bank details) and warrants a stronger enforcing of data privacy policies.

### Other applications

AI and big data can play a significant role in enhancing monitoring capabilities in aspects of MS care/research other than motor and cognitive functioning. These range from passive monitoring of sleep and heart rate variability to the analysis of big data from real-world clinical records. We have grouped these different topics in a single encompassing section, given the small number of publications available thus far, to discuss their potential contribution towards further advancing the standard of care for pwMS, as well as their limitations.

Woelfle et al. (48) recruited 31 pwMS and 31 HCs, with the aim of studying whether remote monitoring of heart rate and sleep parameters could complement step count data in explaining MS severity. Participants wore a commercially available smartwatch (Fitbit Versa 2) for six weeks, during which parameters were extracted for sleep (e.g., sleep efficiency, light/deep/REM sleep duration), heart rate, and activity(e.g., proportion of sedentary/lightly active/fairly active/very active). While activity measures were predictably those most strongly correlated with clinical scales of disability and gait tests, median heart rate and deep sleep proportion also showed moderate correlations. Moreover, incorporating sleep and heart rate measures increased the ability to predict disability (measured by EDSS score), compared to using either baseline sociodemographic data and/or smartwatch-derived motor parameters. This pilot study with a small sample size suggests that sleep and heart rate data may indeed complement activity measures in explaining disease severity. These results are encouraging, especially for the promised ability to track objective sleep parameters remotely and through minimally invasive and economical devices, as compared to portable EEGs or polysomnography performed in the lab, greatly enhancing the feasibility of longitudinal studies of sleep. However, the small sample size warrants further larger studies, to increase the generalizability of results, especially since smartwatch data was lost for 7/62 participants due to synchronization issues, highlighting the need for more reliable data storage and synchronization technologies before such tools can be deemed reliable for larger clinical trials.

Hilty et al. (49) used a previously validated and CE-certified wearable for heart rate detection, with the aim of studying the autonomic nervous system in 56 pwMS and 26 HCs, by analysing circadian trends recorded continuously over a period of two weeks. They applied signal processing algorithms and polynomial regression algorithms to reconstruct circadian trends from big data acquired continuously at 1Hz by the sensor. They observed that circadian trends could distinguish not only pwMS from HCs, but also between pwMS with/without evidence of inflammatory activity (defined either by radiological activity or by a clinical relapse in the prior 12 months), between those with/without evidence of disease progression (defined by neurological deterioration without a relapse event), and between those with low/moderate-to-high disability (defined using an EDSS score cutoff = 3). Their results suggest that continuous heart rate monitoring could enable to uncover specific circadian patterns which distinguish pwMS across inflammatory states (associated with overactive sympathetic activity at night and overall reduced circadian variability) and disease progression (associated with overall reduced heart rate variability and reduced circadian adaptation of the autonomic nervous system). Therefore, autonomic nervous system monitoring with wearable sensors could provide new digital biomarkers and serve as an endpoint in clinical trials for both immunoregulation and symptomatic treatment. Notably, at least seven days of continuous wearing were required to establish robust circadian trends due to high variability of wearable-based heart rate at both the intra-individual and inter-individual level. More studies on larger and more heterogeneous cohorts are needed to confirm these results and increase the generalizability of these results, as >80% of this sample was made up of people with RRMS.

Seccia et al. (50) focused on the application of AI to analyse real-world clinical records of 1624 pwMS (totalling over 18,000 records between 1978 and 2018). They tried to predict the probability of shifting from the relapsing-remitting to the progressive phase at different timepoints (180, 360, 720 days from last visit). They tested predictions based on data from the last available visit using different ML models (visit-oriented approach), or based on the entire clinical history (history-oriented approach) using a specifically designed recurrent neural network (RNN). They found that the visit-oriented approach was better at predicting shifts to progressive MS at 180 days, largely thanks to the inclusion of imaging and liquor history, suggesting that these two methods are informative on the risk of conversion to progressive MS in the short term. Conversely, the history-oriented approach performed better for predictions of shifting to progressive MS at longer intervals (360 and 720 days), owing largely to its better precision (reflecting less false positives). Crucially, the history-oriented approach was more reliant on clinical features, as both MRI and liquor data was unavailable for the majority of participants at all time points. Taken together, these results indicate that AI can effectively leverage real-world clinical big data to predict the risk of conversion to progressive MS. One key limitation is the intrinsic nature of real-world clinical data, which often contains missing data, as seen for liquor and MRI data in this study. It is crucial that clinical expertise is applied during the planning of analysis and data preprocessing, to determine if missing data are meaningful or not, and how they should be dealt with (e.g., missing liquor data can be expected, as lumbar punctures are not performed at each clinical visit, whereas EDSS score should ideally be available at all timepoints). This once again underlines the importance of data collection and maintenance. A well-structured and well-described feature set allows for much easier collaborations and sharing of data, thus promoting the fusion of different expertise (namely clinical and data science), which could further increase our understanding of MS. Accurate data maintenance could also allow to perform future analyses on data with longer follow-up durations, increasing our understanding of longitudinal disease patterns in MS.

## Conclusion

The growing adoption of digital remote monitoring tools has great potential to improve both research and clinical aspects of MS, thanks to remote tracking of motor and non-motor symptoms. This review highlights that connected devices like smartphones and, especially, wearables can effectively monitor motor impairments, such as fall risk and gait disturbances, through continuous, granular data collection during real-world activities. Remote monitoring of physical activity is gaining significant traction in clinical research application. This is demonstrated by the inclusion of remote activity monitoring data as an exploratory endpoint in a recent drug trial (51), albeit through a basic daily step count metric. Further improvements may derive from AI algorithms which can recognize activity states, enriching the quantitative sensor data.

The evidence available on cognitive monitoring still favours the adaptation of active cognitive tests in digital form, to allow remote longitudinal monitoring, which may increase the standard of care for those with reduced mobility and/or access to specialized MS care. Recent advances in AI-driven cognitive tests and keystroke big data provide potential pathways to enable passive cognitive monitoring, but further research is needed to confirm their reliability and clinical utility.

Some studies have explored less-studied domains like sleep and circadian autonomic patterns, with interesting results which suggest that remote monitoring of these domains is feasible and could provide novel insights, compared to traditional research methods. Finally, preliminary exploratory studies have leveraged big data from clinical health records, with promising results, highlighting the need for careful recording, structuring, and maintenance of real-world clinical datasets. Increased awareness of the importance of big data in MS has led to the rising prominence of collaborative databases, both on a national (52–54) and international scale (55, 56), as well as multicentric studies on digital outcomes (57).

However, despite these advancements, challenges remain, including the small sample sizes observed in many studies, which limit the generalizability of their results to different MS populations, namely those with progressive MS, higher disease severity, and reduced access to specialized MS centres. Inclusiveness is a key area which should be addressed more carefully by future studies. Indeed, when assessing the real-world feasibility of digital monitoring for the entire MS population, researchers should be mindful of potential sampling bias, as patients willing/able to undergo such protocols may present distinct features (e.g., younger patients, with lower disability, higher educational attainment, and without cognitive impairment). For the use of AI and ML algorithms, researchers should never forget that an algorithm with many input variables may be very accurate but unusable by non-specialized MS centres which cannot obtain all the clinical/instrumental/sensor data on which the algorithm was trained on. Another significant limitation is the heterogeneity of monitoring methods and study protocols, which negates the possibility to compare feasibility, reliability, and validity data across different studies and devices. Future studies should strive to address these outstanding issues, since feasible, reliable and valid digital monitoring tools represent an invaluable resource for both research and clinical practice.

Finally, the recent rise and diffusion of conversational AI agents (e.g., ChatGPT) has led to some researchers exploring their usefulness in the setting of MS care (58, 59). When applied to disease monitoring, conversational AI could be integrated in eHealth apps as a chatbot, similar to examples from other fields (see for example (60)). This could allow patients to report their symptoms in a conversational manner, instead of having to answer omni-comprehensive and pre-defined structured lists of questions or questionnaires, which could feel alienating and repetitive, leading to low adherence. This may not only be perceived as a more natural and interpersonal approach by patients, but may also reduce their burden, by eliminating the need to answer questions which are not relevant for them at that moment in time. Moreover, an AI-driven closed loop system may also guide the administration of validated patient-reported questionnaires through eHealth apps, by selecting only the questionnaires that are most relevant for each individual patient, based on their reported symptoms at that specific timepoint. We hypothesize that this approach would reduce the time and energy demand on patients, while also providing a more interpersonal, responsive and adaptive monitoring framework, which could then lead to higher adoption and adherence to digital long-term monitoring. However, systematic studies are required to substantiate these hypotheses. Firstly, studies should evaluate the technical feasibility of applying conversational AI to longitudinal symptoms monitoring in MS, focusing particularly on the safety, validity and reliability of the information provided by AI. Secondly, they should investigate the expectations and needs of patients, caregivers and clinicians toward digital monitoring, to determine if and how AI can be applied to address them.
